# Neonatal umbilical cord blood bilirubin as a biomarker for hyperbilirubinemia required phototherapy

**DOI:** 10.6026/973206300211677

**Published:** 2025-06-30

**Authors:** Madhav Shridharrao Kadam, Mohit Garg, Seema Sutay, Sandeep Singh Matreja, Priyadarshini Rangari, Amit Rangari

**Affiliations:** 1 Department of Biochemistry, Parbhani Medical College and RP Hospital Research Institute, Maharashtra, India; 2 Department of General Medicine, Nandkumar Singh Chouhan Government Medical College, Khandwa, Madhya Pradesh, India; 3 Department of Forensic Science, Nandkumar Singh Chouhan Government Medical College, Khandwa, Madhya Pradesh, India; 4 Department of Pathology, Nandkumar Singh Chouhan Government Medical College, Khandwa, Madhya Pradesh, India; 5 Department of Dentistry, Nandkumar Singh Chouhan Government Medical College, Khandwa, Madhya Pradesh, India; 6 Department of Microbiology, Nandkumar Singh Chouhan Government Medical College, Khandwa, Madhya Pradesh, India

**Keywords:** Cord blood bilirubin, neonates, neonatal hyperbilirubinemia, phototherapy, serum total bilirubin

## Abstract

Jaundice is clinical condition characterized by transient deficiency of bilirubin conjugation, leading to neonatal hyperbilirubinemia. Therefore, it is of interest to assess cord blood bilirubin as a predictor for neonatal hyper-bilirubinemia (HB) needing phototherapy in full-term neonates. In 220 neonates with HB were assigned as cases and without HB as controls. The mean cord blood bilirubin level was 2.64±0.63 and total serum bilirubin estimated on 3rd day of life was 16.14±1.4. Umbilical cord blood bilirubin estimation is a non-invasive, economical and simple method of predicting subsequent neonatal hyper-bilirubinemia that can help clinicians for early discharge of normal neonates and follow-up in high-risk infants.

## Background:

Jaundice is a common reason for neonatal therapy and is caused by a transitory decrease in the liver's conjugation ability. Serum total bilirubin within the physiological range indicates a balance in bilirubin excretion and synthesis in newborns. However, in certain new-borns, these two processes are out of balance, resulting in hyperbilirubinemia [1]. Nearly 80% of preterm newborns and 60% of term neonates have hyperbilirubinemia, which has been linked to neuronal impairment that affects quality of life. In the Indian healthcare system, there is a trend of early release of healthy full-term babies due to economic considerations and a lack of resources [2]. Many of these new-borns are readmitted to the NICU for hyperbilirubinemia treatment, which necessitates phototherapy, adding to the cost burden on families and treating institutions. These readmissions expose healthy newborns to extremely infectious hospital surroundings, cause emotional disruptions in mothers and interfere with regular nursing habits [3]. Before releasing the newborn, the risk of substantial hyperbilirubinemia is constantly assessed, including transcutaneous bilirubin, clinical assessment of risk factors and total blood bilirubin. Neonates released within 48 hours of delivery must be seen by a paediatrician within 2-3 days, according to American Academy of Paediatrics [4]. Therefore, it is of interest to assess cord blood bilirubin at birth as a predictor of significant neonatal hyperbilirubinemia needing phototherapy in full-term neonates.

## Materials and Methods:

The present prospective clinical study was conducted at Nandkumar Singh Chouhan Government Medical College, Khandwa and Madhya Pradesh. Verbal and written informed consent was taken from all the subjects before participation. The study included healthy term neonates from 37-42 weeks of gestation as assessed using the New Ballard score, neonates from both genders that were delivered by either cesarean or vaginal delivery with birth weight of ≥2500 grams. The exclusion criteria for the study were subjects with gestational age <37 weeks, low birth weight babies, perinatal hypoxia, sepsis and subjects with major congenital anomalies. Also, a 2ml cord blood sample was collected from all the newborns from the placental side of the cord during delivery in a red color-coded vacutainer. IT was ensured that samples were not exposed to the light while transporting these samples to the laboratory and during their storage and processing.

Hemolyzed blood samples were excluded and bilirubin was evaluated using the Daizo method. Total serum bilirubin measurement was repeated on 3rd day with serum samples obtained by venipuncture procedure. The study included 220 neonates with 110 subjects with hyperbilirubinemia needing phototherapy as cases and 110 neonates without hyperbilirubinemia as controls. The data gathered were analyzed statistically using SPSS software version 24.0 for assessment of descriptive measures, Student t-test, ANOVA, Pearson correlation coefficient and Chi-square test. The results were expressed as mean and standard deviation and frequency and percentages. The p-value of <0.05 was considered.

## Results:

There were 56% (n=112) males and 44% (n=88) females in the present study. 110 neonates had hyperbilirubinemia and needed phototherapy and 110 neonates had no hyperbilirubinemia. Rh incompatibility was seen in 14% (n=14) subjects and ABO incompatibility was seen in 6% (n=6) subjects. The mean APGAR score in study subjects was 7.82 and the mean birth weight was 2.37 kg. The study results showed that for comparison of umbilical cord bilirubin (UCB) and 3rd-day bilirubin levels in neonates requiring phototherapy, in subjects requiring phototherapy, the mean value of umbilical cord blood was 2.64±0.656 and 3rd-day total bilirubin was 16.14±1.681 showing statistically significant results with p=0.001.

In subjects that did not need phototherapy, the mean umbilical cord blood value was 1.64±0.511 and 3rd-day total bilirubin was 9.90±2.057. The difference was highly statistically significant with p=0.001. It was seen that for assessment of the correlation of UCB and 3rd-day TB in study subjects, the r- value in subjects needing phototherapy was 0.085 and in subjects that did not need phototherapy, r- value was-0.023. The results were statistically non-significant for both I n subjects that needed or did not need phototherapy with p=0.546 and 0.863 respectively Table 1 (see PDF). It was also seen that for specificity and sensitivity of 3rd day total bilirubin and umbilical cord bilirubin in prediction of hyperbilirubinemia, for UCB, were shown in Table 2 (see PDF).

## Discussion:

The mean APGAR score in study subjects was 7.82 and the mean birth weight was 2.37 kg. These data were comparable to the studies of Patil *et al.* [3] in 2024 and Reddy *et al.* [4] in 2021. In the subjects requiring phototherapy, the mean value of umbilical cord blood was 2.64±0.656 and 3rd-day total bilirubin was 16.14±1.681 [statistically significant p=0.001]. In subjects that did not need phototherapy, the mean umbilical cord blood value was 1.64±0.511 and 3rd day total bilirubin was 9.90±2.057 [highly statistically significant p=0.001]. These results were consistent with the findings of Keren *et al.* [5] in 2005 and Vasudevan *et al.* [6] in 2013. The correlation of UCB and 3rd day TB in study subjects, was statistically non-significant for both groups with p=0.546 and 0.863 respectively and also similar to the results of Gupta *et al.* [7] in 2021 and Rehna *et al.* [8] in 2021. The specificity and sensitivity of 3rd day total bilirubin and umbilical cord bilirubin in prediction of hyperbilirubinemia, for UCB, results correlated with the findings of Zeitoun *et al.* [9] in 2013 and Bernaldo *et al.* [10] in 2004.

## Conclusion:

A cut-off value of 2.5mg/dL in cord blood bilirubin can be used for the prediction of significant neonatal hyperbilirubinemia needing phototherapy in full-term neonates. Also, umbilical cord blood bilirubin estimation is a non-invasive, economical and simple method of prediction of subsequent neonatal hyperbilirubinemia that can help clinicians for early discharge of normal neonates and follow-up in high-risk infants.
